# Single-nucleotide polymorphisms(SNPs) in a sucrose synthase gene are associated with wood properties in *Catalpa fargesii* bur

**DOI:** 10.1186/s12863-018-0686-8

**Published:** 2018-11-01

**Authors:** Nan Lu, Fang Mei, Zhi Wang, Nan Wang, Yao Xiao, Lisheng Kong, Guanzheng Qu, Wenjun Ma, Junhui Wang

**Affiliations:** 1 State Key Laboratory of Forest Genetics and Tree Breeding, Key Laboratory of Tree Breeding and Cultivation of State Forestry Administration, Research Institute of Forestry, Chinese Academy of Forestry, Beijing, 100091 People’s Republic of China; 2 Department of Biology, Centre for Forest Biology, University of Victoria, 3800 Finnerty Road, Victoria, BC Canada; 3 State Key Laboratory of Tree Genetics and Breeding, Northeast Forestry University, 26 Hexing Road, Harbin, 150040 People’s Republic of China

**Keywords:** Association study, Single-nucleotide polymorphism, Linkage disequilibrium, Sucrose synthase, Wood properties, *Catalpa fargesii* bur

## Abstract

**Background:**

Association study is a powerful means for identifying molecular markers, such as single-nucleotide polymorphisms (SNPs) associated with important traits in forest trees. *Catalpa fargesii* Bur is a valuable commercial tree in China and identifying SNPs that associate with wood property would make a foundation of the marker-assisted breeding in the future. However, related work has not been reported yet.

**Results:**

We cloned a 2887 bp long sucrose synthase (*SUS*) gene from the genome of *C. fargesii*, which is a key enzyme in sucrose metabolism and also associated to wood formation in trees, coding 806 amino acids that expressed mainly in young branches, xylem, and leaves according to real-time quantitative PCR. Then we identified allelic variations of *CfSUS* associated with nine wood quality associated traits in *Catalpa fargesii* Bur. Totally, 135 SNPs were identified through cloning and sequencing the *CfSUS* locus from a mapping population (including 93 unrelated individuals) and 47 of which were genotyped as common SNPs (minor allele frequency > 5%) in the association population that comprised of 125 unrelated individuals collected from main distribution area. Nucleotide diversity and linkage disequilibrium (LD) analysis showed *CfSUS* has a relative low SNP diversity (*π*_T_ = 0.0034) and low LD (r^2^ dropped below 0.1 within 1600 bp). Using the association analysis, we found 11 common SNPs and 14 haplotypes were significantly associated with the traits (false discovery rate, Q<0.1), explaining 3.21–12.41% of the phenotypic variance. These results provide molecular markers above associated with wood basic density, pore rate, and six other traits of wood, which have potential applications in breeding of *Catalpa fargesii* Bur.

**Conclusion:**

We first cloned a *SUS* gene in *C. fargesii*, then identified several SNPs and haplotypes that associated with wood properties within this gene, suggesting *CfSUS* participates in the wood formation of *C. fargesii*. Moreover, molecular markers we identified in this study may be applied into marker-assisted breeding of *C. fargesii* in the future.

**Electronic supplementary material:**

The online version of this article (10.1186/s12863-018-0686-8) contains supplementary material, which is available to authorized users.

## Background

*Catalpa fargesii* Bur. is a valuable tree native to China. Its timber not only exhibits good mechanical properties, such as stiffness and ultimate stress, but also has good chemical properties and high corrosion resistance [1]. The properties of wood vary depending on the source material, regardless, improved mechanical properties are the main targets in *C. fargesii* breeding. Although the mechanisms influencing wood properties are unclear, some researchers have speculated that the specific structure, content, arrangement, and interaction of macromolecules in secondary cell walls may confer unique properties, making wood better suited for different applications [2]. This suggests that variations in wood properties may rely on variations in genes involved in the synthesis of lignin, cellulose, hemicellulose, and other components. However, few genes that directly affect wood quality in forest trees have been identified due to the long lifecycle and lack of effective method to get the mutant for both forward and reverse genetics study [2, 3].

Molecular marker-assisted selection has been proven effective in resolving the complex quantitative traits of genetic components to improve and accelerate traditional tree breeding. In particular, association mapping, is an effective way for elucidating the potential relationship between allelic variation and complex quantitative trait variations in natural populations [4]. Previous researches have suggested that association mapping is a useful tool to identify allelic variations within candidate functional genes associated with quantitative traits, which could influence growth, wood properties, and biotic and abiotic resistance, suggesting that association mapping may be applicated in forest tree breeding [5] and in fact, several studies have focused on it. For example, the associations between four significant single-markers in the *PtoPsbW* gene and five wood quality traits have been identified in *Populus tomentosa* [6]. In addition, Fahrenkrog et al. found 23 SNP associations from 22 genes in *Populus deltoides* significantly influenced eight composite traits that associated with wood properties, including lignin percentage, lignin syringyl/guaiacyl ratio, lignin structure, and the growth of plants [7].

Sucrose is the main source of carbon for compounding cellulose, one of the major components of secondary cell walls, and wood vascular tissue in the stem comprises highly active sink cells that use sucrose for cellulose synthesis [8]. In these cells, sucrose synthase catalyses the synthesis of UDP-glucose, which is an immediate precursor of cellulose biosynthesis [9]. *SUS* participates in providing UDP-glucose for cellulose biosynthesis and directly associated with cellulose synthase complexes [10]. Therefore, clarifying the nucleotide diversity and allelic effects of the gene encoding *SUS* may help identify molecular markers associated with wood quality traits to guide *C. fargesii* breeding.

In this study, we first cloned and identified a SUS gene family member in *C. fargesii* (*CfSUS*). Real-time quantitative PCR (RT-qPCR) was used to identify the gene expression in six different organs. Subsequently, nucleotide diversity and linkage disequilibrium decay within the *CfSUS* were assessed in a mapping population (*n* = 93). Finally, single-marker- and haplotype-based association tests were performed to examine the putative effects of allelic variations on nine wood quality traits in an association population comprising 125 *C. fargesii* individuals. This is the first time that association analysis about *CfSUS* were studied to identify molecular markers probably affecting wood property, which could support the genetic improvement of *C. fargesii* and other tree species.

## Methods

### Description of association population

We used a population consisting of 125 unrelated *C. fargesii* individuals that collected from all of the provenances throughout its whole natural distribution range in China, for the initial SNP association mapping. The distribution zone from which these individuals were collected, could be divided into four geographic regions: Fenhe River Valley, Jinghe River Valley, Jialingjiang River Valley, and Yellow River Valley. In the year 2009, branch segments of 125 native *C. fargesii* individuals were collected from eight cities in four provinces and grafted, and the population was grown in Xiaolongshan National Nature Reserve, Gansu Province, China (33°40′N, 106°23′E). The clonal plantation was established using a randomised complete block design with two plants per clone in each block (row spacing is 2 m and plant spacing is 2 m) and totally six replicates. Within the association population, 93 individuals were randomly selected (for each location, at least one *C. fargesii* individual were selected) to identify SNPs within the gene via PCR amplification and sequencing.

### Phenotypic data

The 125 individuals of the association population were sampled in 2012 to characterise their wood quality traits. Cores from bark to pith were collected in the south-facing direction of the original stems to measure wood density and other wood properties with an increment borer (7 mm) at breast height (1.3 m above the ground). The wood samples were fixed in formalin–acetic acid–alcohol (FAA) after collection, and nine wood property traits were measured in 2013: wood basic density (WBD), pore rate, cell wall percentage (the percentage of cell wall in whole cells), cell wall thickness, radial lumen diameter, chordwise lumen diameter, radial fibre central cavity diameter, chordwise fibre central cavity diameter, and average fibre central cavity diameter.

WBD was measured according to the formula WBD = W_2_/(W_1_ − W_2_ + W_2_/ρ_cw_), where W_1_, W_2_ and ρ_cw_ represent the water-saturated weight, oven dry weight, and wood cell wall component density, respectively. We used the constant 1.53 g/cm^3^ for ρ_cw_ [11, 12]. The other wood properties were detected according to Li et al. [13]: Cores from the xylem to pith were split in 3-cm segments and cross-sections (10–15-μm-thick) were prepared using a sliding microtome (Leica, Heidelberg, Germany), stained with 1% safranin, and fixed with Eukitt (Bio-Optica, Milan, Italy) on an object glass. To measure the wood microstructure characteristic parameters, a digital image processing system combining a light microscope (80i, Nikon), video camera sensor (Penguin 600CL; Pixera Corp., Santa Clara, CA, USA), and TDY-5.2 colour image analysis system was used [14]. The phenotype data are listed in Additional file 1: Table S1 and the frequency distributions of all traits could be found in Additional file 2: Figure S1. We selected these nine wood property traits because these traits may influence the final mechanical properties of wood productions [13].The mean, maximum, minimum values and coefficient of variation of the nine phenotypic traits were calculated using SPSS software (ver. 18.0; SPSS Inc., Chicago, IL, USA) and are listed in Additional file 3: Table S2.

### cDNA isolation and genomic DNA amplification of *CfSUS*

Xylem were collected by scraping the thin and partially lignified layer on the exposed xylem surface from the branches of a 1-year-old “Xianhuiqiu” clone. The tissue was frozen immediately in liquid nitrogen and stored in the laboratory at − 80 °C for later RNA extraction. Total RNA was extracted using a RNeasy Plant Kit (Qiagen, Shanghai, China) according to the manufacturer’s instructions. First-strand cDNA was synthesised from 2 g RNA using PrimeScript™ 1st Strand cDNA Synthesis Kit (TaKaRa, Tokyo, Japan). We obtained the 2887-bp complete coding sequence(CDS) of *CfSUS*, including a 2418-bp open reading frame (ORF) from a previous RNA sequencing data. We amplified *CfSUS* cDNA using *SUS*-CDS-specific primers (Additional file 4: Table S3).

Total genomic DNA was extracted from young leaves of a 1-year-old “Xianhuiqiu” clone with the DNeasy Plant Kit (Qiagen). Four specific primers (SUS-a, SUS-b, SUS-c, and SUS-d) were designed to sequence the introns in *CfSUS* based on the cDNA sequence on the conserved domain (Additional file 3: Table S2). After PCR amplification, four fragments were linked to the clone vector T-Vector pMD19 (TaKaRa) and sequenced, and the entire DNA sequence of *CfSUS* was obtained according to the assemblage results of the four sequenced fragments with DNAMAN software (Lynnon Biosoft, Vaudreuil, Quebec, Canada). Finally, 5067 bp genomic DNA sequences of *CfSUS*, including a 1394-bp 5′UTR, 3406-bp coding region, and 267-bp 3′UTR were obtained. The entire DNA sequence of *CfSUS* was verified using the SUS-e primers (Additional file 3: Table S2). The *CfSUS* sequence is deposited in GenBank under the accession number MH394454.

### *CfSUS* and phylogenetic analyses

Amino acid sequences of *CfSUS* was used for BLAST in the GenBank database and multiple *SUS* proteins from other species were selected for phylogenetic tree construction using MEGA software and alignment with DNAMAN software. We analysed the phylogenetic relationship of *CfSUS* with the amino acid sequences of *SUS* from other species identified from NCBI (http://www.ncbi.nlm.nih.gov) using BLAST (Altschul et al. 1997): *Orobanche ramosa* (AEN79500.1), *Nicotiana tabacum* (AHL84158.1), *Solanum tuberosum* (NP_001274911.1 and NP_001275286.1), *Solanum lycopersicum* (CAA09681.1 and ADM47608.1), *Cichorium intybus* (ABD61653.1), *Actinidia chinensis* (AFO84090.1), *Camellia sinensis* (AHL29281.1), *Gossypium hirsutum* (ADY68848.1), *Gossypium barbadense* (ADY68844.1), *Arachis hypogaea* var. vulgaris (AEF56625.1), *Jatropha curcas* (AGH29112.1), *Gossypium aridum* (AEN71079.1), *Gossypium tomentosum* (AEN71067.1), *Eucalyptus grandis* (ABB53602.1), *Populus tomentosa* (ADW80558.1), *Manihot esculenta* (ABD96570.1), *Hevea brasiliensis* (AGQ57012.1, AGM14948.1, and AGM14949.1), *Hordeum rulgare* (CAA46701.1 and CAA49551.1), *Lolium perenne* (BAE79815.1), *Triticum aestivum* (CAA04543.1 and CAA03935.1), *Bambusa oldhamii* (AAV64256.2, AAL50571.1, AAL50570.1, and AAL50572.2), *Oryza sativa* (CAA46017.1, CAA41774.1, and AAC41682.1), *Saccharum officinarum* (AAF85966.1), *Zea mays* (AAA33514.1), and *Tulipa gesneriana* (CAA65639.1). Phylogenetic tree was conducted using MEGA ver. 5(maximum likelihood method) [15]. The statistical confidence of the nodes of the tree was based on 1000 bootstrap replicates.

### *CfSUS* expression in different tissues

Total RNA was extracted from six different tissues, including leaf, bark, phloem, xylem, flower, and young branch, from three 11-year-old “Xianhuiqiu” clone (as three times repetition) using RNeasy Kits (Qiagen, Duesseldorf, Germany) and reverse-transcribed into cDNA using PrimeScript™ 1st Strand cDNA Synthesis Kit (TaKaRa, Tokyo, Japan). The cDNA samples were used to analysis the expression of *CfSUS* in different tissues using RT-qPCR.

RT-qPCR was performed with a LightCycler 480 System (Roche, Basel, Switzerland) using the SYBR Premix Ex Taq Kit (TaKaRa, Tokyo, Japan) with the recommended amplification system by the manual. The primers for amplification (SUS-q; Additional file 3: Table S2) were designed using Primer Express 5.0 software (Applied Biosystems, Life Technologies, New York, NY, USA), and a primer pair of an actin gene (Additional file 3: Table S2) was selected as internal control according to Jing et al. [16]. The PCR program was performed according to the recommended program in the LightCycler 480 System manual, as follows: initial denaturation at 95 °C for 30 s; 40 cycles of 5 s at 95 °C and 30 s at 60 °C; and then one cycle of 5 s at 95 °C, 60 s at 60 °C, and 95 °C (acquisition mode, continuous; acquisitions, five per degree Celsius). Four technical replicates and three biological replicates were performed for all experiments and the results obtained for the different tissues were standardised to the levels of actin using the 2^−ΔΔCT^ method.

### SNP discovery and genotyping

To identify SNPs in the *CfSUS* gene (without considering insertions/deletions), DNA sequences including 53-bp 3′UTR and 3406-bp coding region, was cloned, sequenced, and analysed in 93 randomly selected individuals from the *C. fargesii* association population. To ensure the sequencing accuracy, four pairs of primers (SUS-1, SUS-2, SUS-3, and SUS-4) were used to amplify four fragments (800–1500 bp) of the whole sequence (Additional file 3: Table S2) by PCR using Takara Ex Taq (TaKaRa, Tokyo, Japan) and ligated to pMD™18-T vector (TaKaRa, Tokyo, Japan). Eight clones for each fragment were randomly selected for sequencing. The four fragments were used to assemble the complete *CfSUS* sequence. DNAMAN and ClustalX2 were used for the sequence alignment. The 93 genomic clones were aligned and compared using MEGA ver. 5.0 and DnaSP v5 to identify SNPs and analyse nucleotide polymorphisms [17]. Subsequently, common SNPs (minor allele frequencies ≥0.05) were genotyped across all 125 DNA samples of the association population.

### Nucleotide diversity and linkage disequilibrium analysis

We used Phase v2.1 software to disambiguate the DNA sequences into haplotypes (10,000 iterations applying the Bayesian Markov Chain Monte Carlo approach) [18] and DnaSP v5 software to calculate the summary statistics of the SNPs. Nucleotide diversity was evaluated using π value [19] and θw value [20], which represent the average number of pairwise differences per site between sequences and the average number of segregating sites, respectively [6].

The decay of LD with the increase of physical distance between SNPs within the candidate region of *CfSUS* was estimated by linear regression analysis of LD using DnaSP v4.90.1. The LD level between the 47 common SNP markers were valued as r^2^ (squared correlation of allele frequencies) using the HAPLOVIEW (https://www.broadinstitute.org/haploview/haploview), where the interval of the parameter varies from 0 to 1. The significance of r^2^ (*P*-values) for all the SNP sites were calculated using 100,000 permutations. Genotypic data of *CfSUS* identified in this population were showed in Additional file 5: Table S4.

### Association mapping

The associated mapping was carried on according to the method of Wang et al. [6]. In the association population (*n* = 125), 47 common SNPs and nine wood properties associated traits were considered, and a mixed linear model (MLM) was selected to fit each SNP-phenotype combination using TASSEL v2.0.1 [21]. We used a mixed linear model described as the following formula: y = μ + *Q*υ + Zu + e. In this formula, “y”, “μ”, “υ”, “u” and “e” respectively represent a vector of phenotype observation, (a vector of) intercepts, (a vector of) population effects, (a vector of) random polygene background effects and (a vector of) random experimental errors. Q is a matrix used to define the population structure by STRUCTURE and Z is a matrix relating y to u. Var(u) = *G* = σ_a_^2^κ with σ_a_^2^ is the unknown additive genetic variance and κ is the kinship matrix. In this model, the Q and K represent the estimated membership probability and pairwise kinship, respectively. The Q matrix was identified based on the population structure pattern (K = 3) within the association population (125 unrelated individuals), which was assessed by STRUCTURE v2.3.1 [22]. The K matrix was obtained by the SPAGeDi ver. 1.2. Positive false discovery rate (FDR) was calculated by QVALUE software [23] and used to correct the multiple testing. The percentage of phenotypic variation (*R*^2^) explained by each SNP was calculated use the formula *R*^2^ = SSt/SST, where SSt and SST represented the variance between genotypes and the total variance, respectively [3].

Haplotype frequencies and haplotype association tests were evaluated and performed using Haplotype Trend Regression software (Golden Helix, Inc., Bozeman, MT, USA) on a three-marker sliding window. The significance of the associations was tested using 1000 permutation. Only haplotypes with a frequency not less than 1% were selected and positive FDR (Q ≤ 0.1) was used to correct the multiple test.

The modes of gene action were defined as the ratio of dominant (*d*) to additive(*a*) effects(|*d*/*a*|) estimated from the least-square means for each genotypic class. The algorithm and formulas used for calculating dominance (*d*) and additive(*a*) effects were described by Du et al. [24]. The values of |*d*/*a*| ≤ 0.5 was defined as additive effects, whereas partial or complete dominance was defined as the values within the range 0.50 < |*d*/*a*| < 1.25, and values of |*d*/*a*| ≥ 1.25 were regarded as overdominance.

## Results

### Cloning CfSUS from *Catalpa fargesii*

**Fig. 1:**
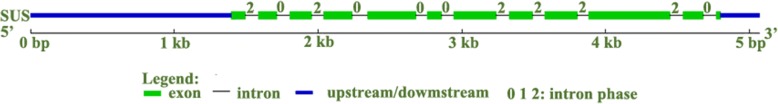
Genomic organisation of *CfSUS*

The full-length cDNA of *CfSUS* was 2887 bp, including a 2418-bp open reading frame (ORF), a 202-bp 5′UTR sequence, and a 267-bp 3′UTR sequence. The full length of the *CfSUS* DNA sequence was 5067 bp, containing a 3406-bp coding region flanked by a 1394-bp 5′UTR sequence and 267-bp 3′UTR sequence (Fig. 1). Alignment of the ORF sequence to the full-length DNA sequence revealed 12 exons and 11 introns in *CfSUS*.

**Fig. 2:**
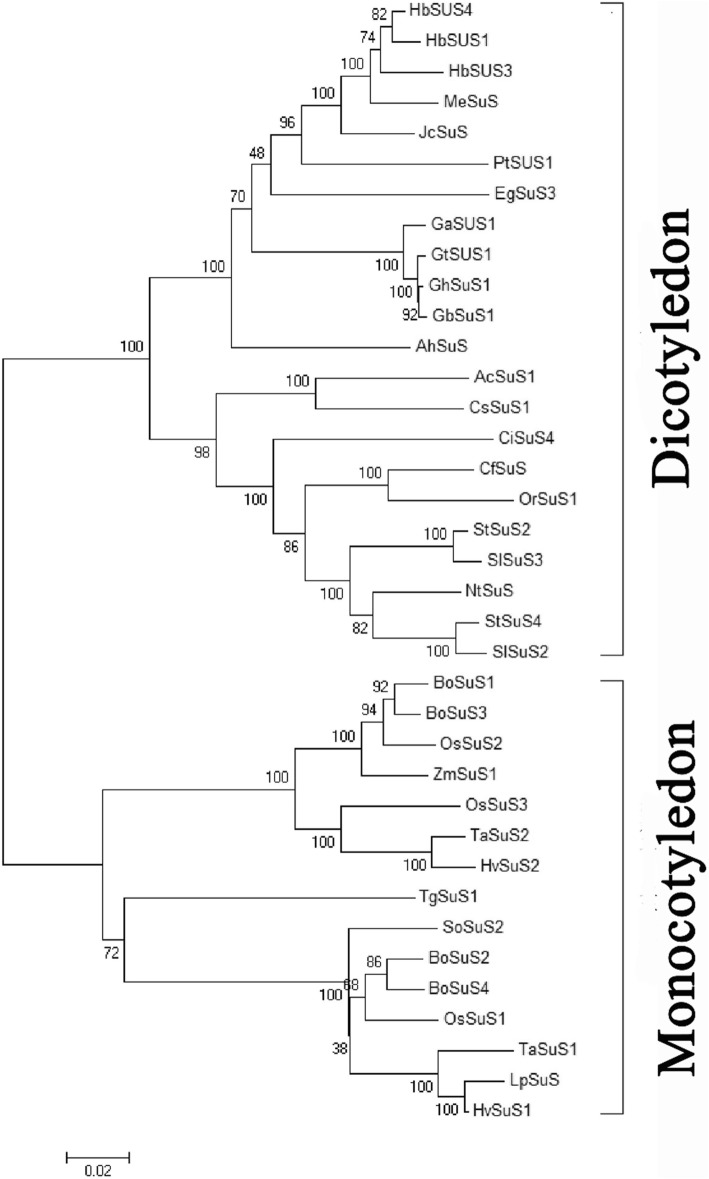
Phylogenetic tree of *SUS* proteins from difference species. *Orobanche ramose* (AEN79500.1): *OrSUS*1; *Nicotiana tabacum* (AHL84158.1): *NtSUS*; *Solanum tuberosum* (NP_001274911.1 and NP_001275286.1): *StSUS*2 and *StSUS*4; *Solanum lycopersicum* (CAA09681.1 and ADM47608.1): *SlSUS2* and *SlSUS3*; *Cichorium intybus* (ABD61653.1): *CiSUS*4; *Actinidia chinensis* (AFO84090.1): *AcSUS*1; *Camellia sinensis* (AHL29281.1):*CsSUS*1; *Gossypium hirsutum* (ADY68848.1): *GhSUS*1; *Gossypium barbadense* (ADY68844.1): *GbSUS*1; *Arachis hypogaea* var. vulgaris (AEF56625.1): *AhSUS*; *Jatropha curcas* (AGH29112.1): *JcSUS*; *Gossypium aridum* (AEN71079.1): *GaSUS*1; *Gossypium tomentosum* (AEN71067.1): *GtSUS*1; *Eucalyptus grandis* (ABB53602.1): *EgSUS*3; *Populus tomentosa* (ADW80558.1): *PtSUS*1; *Manihot esculenta* (ABD96570.1): *MeSUS*; *Hevea brasiliensis* (AGQ57012.1, AGM14948.1 and AGM14949.1): *HbSUS*1, *HbSUS*3, *HbSUS*4; *Hordeum rulgare* (CAA46701.1 and CAA49551.1): *HvSUS*1*, HvSUS*2; *Lolium perenne* (BAE79815.1): *LpSUS*; *Triticum aestivum* (CAA04543.1 and CAA03935.1): *TaSUS*1, *TaSUS*2; *Bambusa oldhamii* (AAV64256.2, AAL50571.1, AAL50570.1, and AAL50572.2): *BoSUS*1, *BoSUS*2, *BoSUS*3, and *BoSUS*4; *Oryza sativa* (CAA46017.1, CAA41774.1, and AAC41682.1): *OsSUS*1, *OsSUS*2, and *OsSUS*3; *Saccharum officinarum* (AAF85966.1): *BoSUS*2; *Zea mays* (AAA33514.1): *ZmSUS*1; *Tulipa gesneriana* (CAA65639.1): *TgSUS*1; *Catalpa fargesii*: *CfSUS*

The molecular phylogeny of *SUS* genes was divided into two groups, dicotyledons and monocotyledons, which indicated that *SUS* gene separation may have occurred after dicotyledons and monocotyledons diverged. *CfSUS* was grouped with other dicots, and the evolutionary tree revealed a closer genetic relationship of *CfSUS* with the *SUS* protein from four Tubiflorae species, *Orobanche ramose*, *Solanum tuberosum*, *Solanum lycopersicum*, and *Nicotiana tabacum*, corresponding to the botanical classification (Fig. 2).

**Fig. 3:**
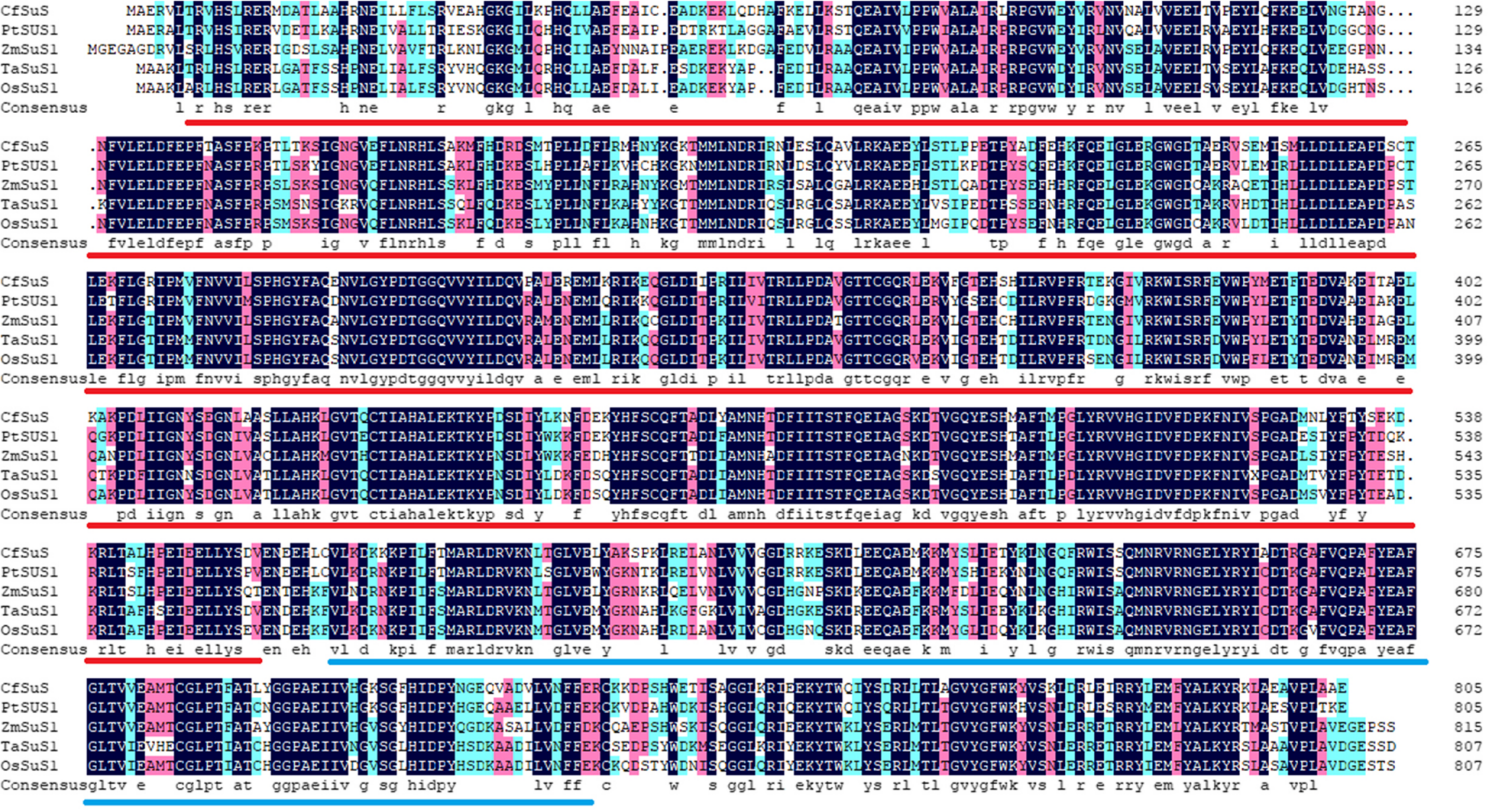
Alignments of *CfSUS* and sucrose synthase from other species. The underlined sections indicate conserved domains, where the red lines indicate the sucrose synthase domain and blue lines indicate the glycosyl transferase domain. *Populus tomentosa* (ADW80558.1): *PtSUS*1; *Zea mays* (AAA33514.1): *ZmSUS*1; *Triticum aestivum* (CAA04543.1 and CAA03935.1): *TaSUS*1; *Oryza sativa* (CAA46017.1): *OsSUS*1; *Catalpa fargesii*: *CfSUS*

Sequence alignment showed that *CfSUS* was similar to *SUS* genes from other species at the amino acid level. For example, *CfSUS* and other *SUS* shared two characteristic functional domains, a glucosyl-transferase domain and a sucrose synthase domain (Fig. 3).

### Tissue-specific *CfSUS* expression

**Fig. 4:**
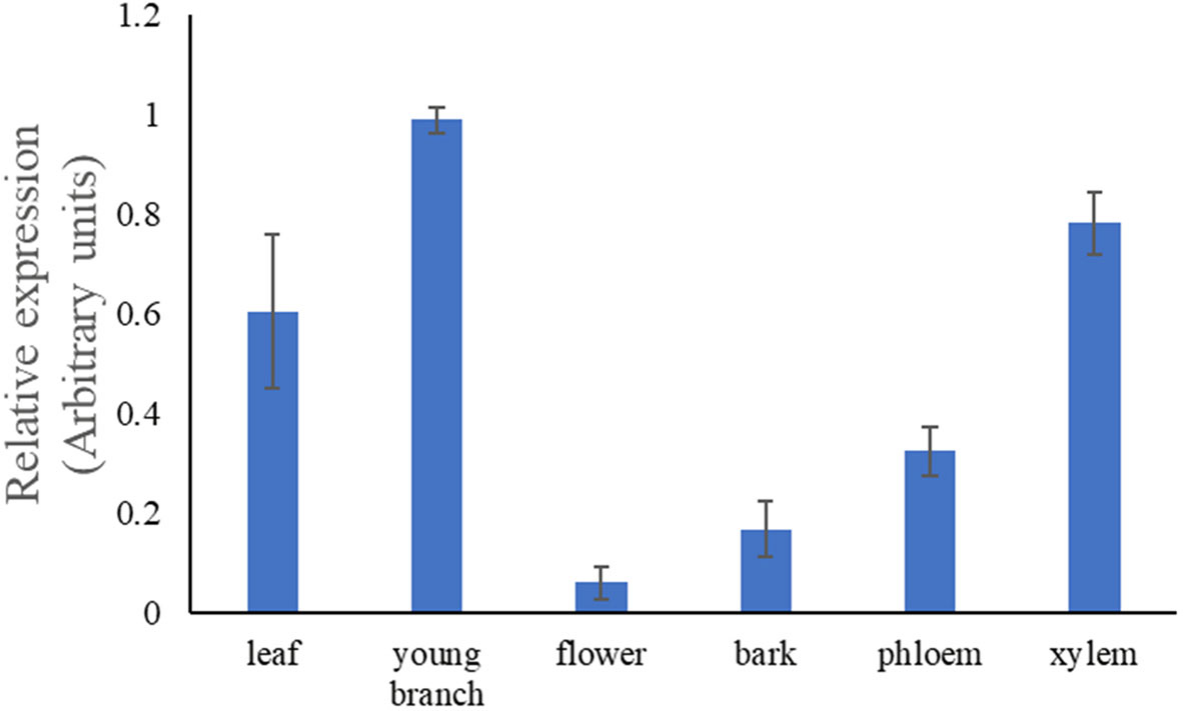
Relative levels of *CfSUS* transcripts in different organs. The error bars represent the standard deviation of three biological replicates

The transcription levels of *CfSUS* were measured in six tissues, including phloem, xylem, leaf, bark, flower, and young branch tissue, with qRT-PCR using designed primers and an actin gene was selected as an internal control gene. The expression of *CfSUS* were detected in all six tissues, resulting different degrees of expression (Fig. 4). Young branches had the highest abundance, followed by xylem. By contrast, flower tissue contained a very low abundance, which may be related to the low lignification of this organ. *CfSUS* expression in young branch and xylem was 3.1 and 2.4 times higher than the expression in phloem. The higher expression in young branch and xylem suggested that *CfSUS* may be involved in cellulose biosynthesis in them.

### Nucleotide diversity and linkage disequilibrium analysis

To characterise the nucleotide diversity and linkage disequilibrium of *CfSUS*, 3459-bp genomic region of *CfSUS*, including 53 bp of 3′UTR, 988 bp of introns and 2418 bp of exons, was amplified and sequenced from 93 individuals in the association population. After defining the phased haplotypes among the 93 unrelated individuals using Phase v2.1, we conducted a more detailed SNP variation analysis in the three regions of *CfSUS* and calculated the nucleotide diversity profiles at these locations (Table 1). Totally, 135 SNPs were identified in the assessed region, with the frequency of 3.93%, based on the aligned sequences of the 93 samples (Table 1). In the coding region, the highest frequency of nucleotide polymorphisms was found in intron 3, while the lowest was found in exons 2 and 12, with no SNP. In total, we found 76 SNPs in exons, of which only 19 SNPs led to synonymous changes and the others were nonsynonymous mutations (Table 1). Of the 135 identified SNPs, 47 (34.6%) were considered to be common (frequency > 0.05) (Additional file 6: Figure S2), and the *CfSUS* locus exhibited low nucleotide diversity (*π*_T_ = 0.0034, *θw* = 0.0078; Table 1). The nucleotide diversity (*π*_T_) ranged from 0 (exons 2 and 12) to 0.0103 (intron 3), while *θw* ranged from 0 (exons 2 and 12) to 0.0241 (intron 3) in coding regions.

**Table 1:** Nucleotide polymorphisms at the *CfSUS* locus

Region	No. of bp	No. of polymorphic sites	Percentage Polymorphism (%)	Nucleotide diversity	
				*π*_T_	*θ*w
Exon 1	98	4	4.08	0.0015	0.0080
Intron 1	93	3	3.23	0.0009	0.0063
Exon 2	128	0	0	0	0
Intron 2	90	3	3.33	0.0061	0.0065
Exon 3	151	4	2.65	0.0022	0.0052
Intron 3	89	9	10.11	0.0103	0.0241
Exon 4	193	2	1.03	0.0002	0.0020
Intron 4	107	7	6.54	0.0037	0.0129
Exon 5	338	11	3.24	0.0024	0.0064
Intron 5	83	3	3.61	0.0022	0.0071
Exon 6	96	1	1.04	0.0015	0.0020
Intron 6	86	4	4.65	0.0025	0.0094
Exon 7	289	13	4.50	0.0026	0.0090
Intron 7	93	3	3.23	0.0043	0.0066
Exon 8	167	4	2.40	0.0016	0.0050
Intron 8	85	6	7.06	0.0099	0.0140
Exon 9	225	10	4.44	0.0035	0.0087
Intron 9	77	7	9.09	0.0051	0.0178
Exon 10	567	21	3.99	0.0027	0.0073
Intron 10	96	6	6.25	0.0051	0.0124
Exon 11	137	6	4.38	0.0027	0.0086
Intron 11	89	3	3.37	0.0026	0.0066
Exon 12	29	0	0	0	0
3′UTR	53	6	11.32	0.0196	0.0226
Total	3459	135	3.93	0.0034	0.0080
Synonymous	–	19	–	–	–
Nonsynonymous	–	57	–	–	–

**Fig. 5:**
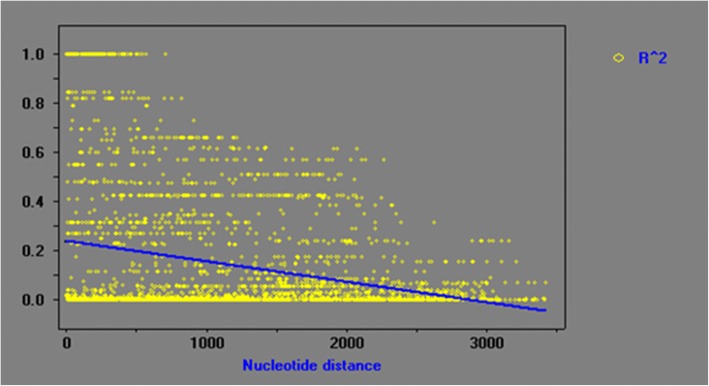
Decay of LD within *CfSUS* based on sequences of the *CfSUS* region from 93 unrelated individuals. Pairwise correlations between SNPs are plotted against the physical distance between SNPs in the sequences. The curves showed the nonlinear regression of r^2^ to the physical distance of the sequence

**Fig. 6:**
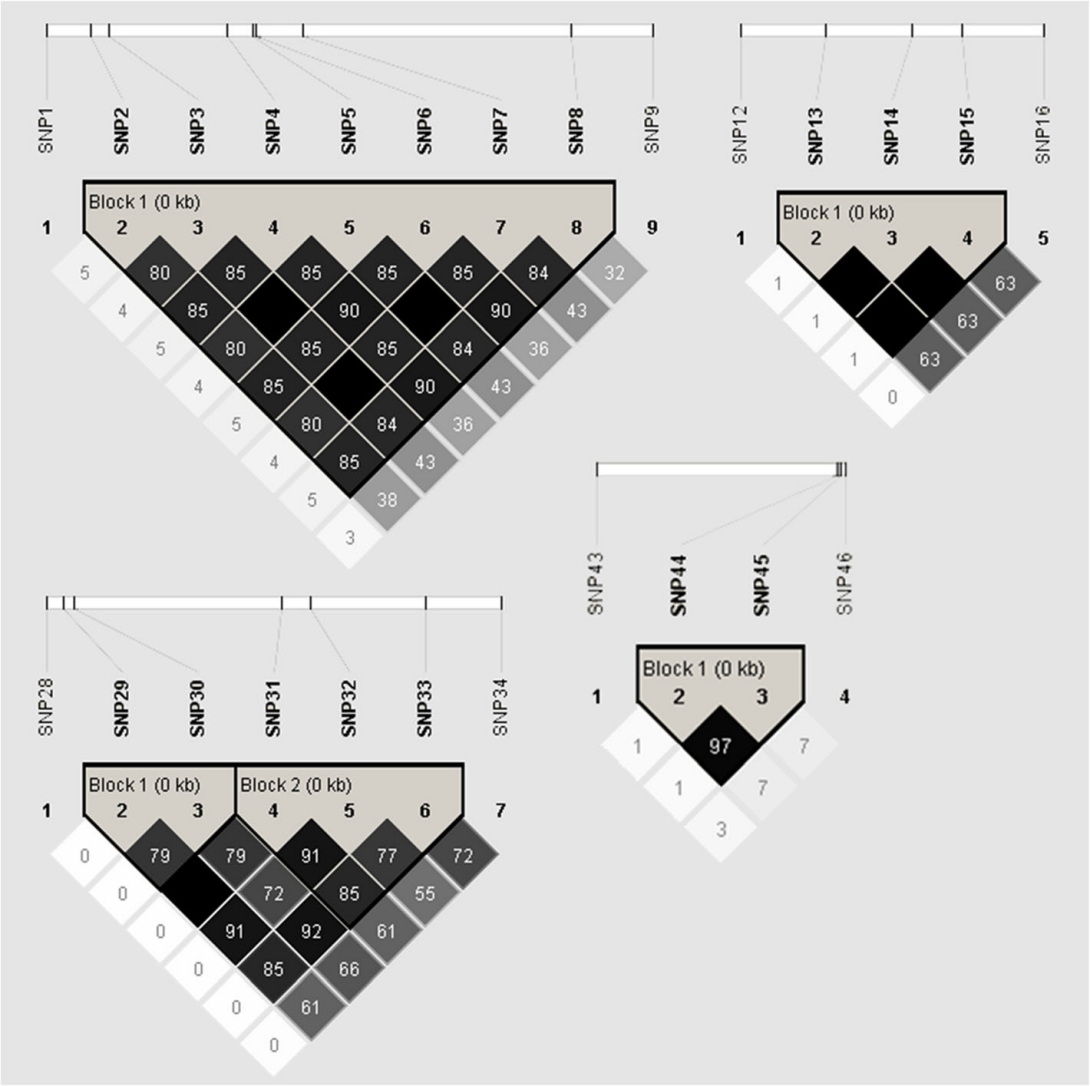
Five distinct haplotype blocks within the *CfSUS* gene. The value of r^2^ is shown by the numbers in the squares. The bold lines represent the relative locations of the SNPs within the gene

The degree of LD showed a linear regression at a relatively rapid rate; the r^2^ value dropped to 0.1 in less than 1600 bp (Fig. 5), indicating that LD may not extend over the entire detected region. Thus, we genotyped 47 common SNPs across 125 individuals and performed LD analysis using the genotype data, which revealed five distinct haplotype blocks within the *CfSUS* gene: SNP 2 to 8, 13 to 15, 29 to 30, 31 to 33, and 44 to 45 (Fig. 6). Overall, LD between the SNPs was high within each block (r^2^ > 0.75).

### Detection of phenotype–genotype associations

We conducted 423 tests (47 common SNPs × 9 traits) using MLM to identify the single-SNP-based associations. In total, 17 significant associations with eight phenotypic traits (excluding cell wall thickness) were identified (*P* < 0.05 and Q < 0.10) (Table 2), including 11 SNPs from five exons (3, 5, 7, 9, and 10) and two intron (3 and 4) regions in *CfSUS*, explained 5.11–12.10% phenotypic variance (Table 2). For the 11 identified SNPs, 4 were noncoding, 4 were synonymous, and 3 were nonsynonymous (Table 2). One of the nonsynonymous marker SNP 9 (arginine to threonine) in exon 5 was significantly associated with WBD (R^2^ = 5.93%). In this case, the value of |***d*/*a*|** was 0.189 and appeared to be additive effect (Table 3). As another nonsynonymous marker, SNP 30 in exon 9 (lysine to threonine) was also significantly associated with WBD (R^2^ = 6.39%) and the mode of gene action was appeared to dominant effects (|*d*/*a*| = 5.636, Table 3). Meanwhile, the synonymous marker SNP 23 in exon 3, associated with radical lumen diameter, showed a difference between the two genotypic classes (19.14 μm in CC and 18.47 μm in CT) (Additional file 7: Figure S3). SNP 16 in exon 7, another synonymous mutation, was associated with chordwise lumen diameter and chordwise central diameter, and explained 7.76% and 5.38% of the phenotypic variance, respectively.

**Table 2:** SNP markers significantly associated with wood quality traits in the association population (*n* = 125)

Traits	Maker	Position	Polymorphism	*P* value	Q value	r^2^(%)
Wood basic density	SNP5	Intron3	[C:T]	0.0363	0.0323	5.62%
	SNP6	Intron3	[C:T]	0.0494	0.0368	5.11%
	SNP9	Exon5	[G:T] ^NS^	0.0302	0.0314	5.93%
	SNP27	Exon9	[A:G]	0.0431	0.0259	5.37%
	SNP30	Exon9	[C:T] ^NS^	0.0227	0.0149	6.39%
Pore rates	SNP37	Exon3	[A:G]	0.0175	0.0427	8.09%
Cell wall percentages	SNP34	Exon10	[A:C]^NS^	0.0355	0.0363	7.34%
Radial lumen diameter	SNP23	Exon3	[A:G]	0.0042	0.0030	9.26%
	SNP34	Exon10	[A:C]^NS^	0.0400	0.0371	7.13%
Chordwise lumen diameter	SNP7	Intron3	[C:T]	0.0336	0.0398	7.47%
	SNP8	Intron4	[G:T]	0.0023	0.0404	12.10%
	SNP16	Exon7	[C:T]	0.0286	0.0352	7.76%
Radial fiber central cavity diameter	SNP23	Exon3	[A:G]	0.0124	0.0386	7.55%
Chordwise fiber central cavity diameter	SNP7	Intron3	[C:T]	0.0481	0.0343	6.86%
	SNP8	Intron4	[G:T]	0.0077	0.0226	10.17%
	SNP16	Exon7	[C:T]	0.0369	0.0420	5.38%
Average fiber central cavity diameter	SNP23	Exon3	[A:G]	0.0124	0.0386	7.55%

*Ns* nonsynonymous polymorphism, *r*^*2*^ percentage of phenotypic variance

**Table 3:** List of marker effects of significant marker–trait pairs

Trait	SNP	2a^a^	d^b^	d/a	2a/S_p_	Frequency	ɑ^c^
Wood basic density	SNP5	−0.046	−0.007	0.304	−1.345	8.0%(T)	−0.00003
	SNP6	−0.027	0.004	−0.296	−0.790	9.2%(T)	−0.00008
	SNP9	0.053	−0.005	−0.189	1.550	9.6%(A)	0.00009
	SNP27	−0.060	0.016	−0.533	−1.755	9.6%(C)	0.00053
	SNP30	−0.011	0.031	−5.636	−0.322	11.2%(C)	0.00031

^a^Calculated as the difference between the phenotypic means observed within each homozygous class (2a = |G_BB_ − G_bb_|, where G_ij_ is the trait mean in the ij^th^ genotypic class)

^b^Calculated as the difference between the phenotypic mean observed within the heterozygous class and the average phenotypic mean across both homozygous classes [d = G_Bb_ − 0.5(G_BB_ + G_bb_), where G_ij_ is the trait mean in the ij^th^ genotypic class]

S_p_: Standard deviation of the phenotypic trait under consideration

^c^The additive effect was calculated as a = p_B_ (G_BB_) + p_b_ (G_Bb_) − G, where G is the overall trait mean, Gij is the trait mean in the ij^th^ genotypic class, and p_i_ is the frequency of the i^th^ marker allele

Of the markers from introns, SNP 5 and SNP 6 in intron 3 were significantly associated with WBD, with small effects ranging from 5.11 to 5.62% (Table 2). Meanwhile, SNP 8 in intron 4 was significantly associated with chordwise lumen diameter and chordwise central diameter and had the same allelic effects in these two traits (Additional file 7: Figure S3). Moreover, there were higher levels of heterozygous trees (TG) of this marker than homozygous trees (GG) (13.65 μm in TG vs. 13.57 μm in GG, and 16.45 μm in TG vs. 16.34 μm in GG, respectively). Similarly, SNP 7 in intron 3 was associated with chordwise lumen diameter and chordwise central diameter.

Haplotype Trend Regression software was used to identify significant associated haplotypes and wood quality traits. In total, ten significant regions, including 14 common haplotypes (frequency > 1%), were significantly associated with seven traits (excluding cell wall thickness and cell wall percentage) (Table 4). Among these, two haplotypes from SNP 35–37 were associated with pore rate, while three haplotypes from SNP 33–35 were associated with WBD, which was supported by the single-SNP associations (SNP 37). In addition, one haplotype from SNP 7–9 was associated with four traits, including radial lumen diameter, chordwise lumen diameter, and chordwise central diameter, and two of the four haplotype-based associations (chordwise lumen diameter and chordwise central diameter) were supported by the previous SNP associations (SNPs 7 and 8). These haplotypes explained 3.21–12.41% of the phenotypic variation.

**Table 4:** Haplotypes significantly associated with wood quality traits

Trait	*p*-value	Q-value	R^2^(%)	Haplotype	Frequency (%)	p(ind)	Mean
Wood basic density	0.040	< 0.1	8.26	SNP19–21			
				T-A-T	6.80%	0.007	0.40
	0.003	< 0.1	12.41	SNP33–35			
				T-A-T	2.00%	0.031	0.39
				T-C-T	6.80%	0.007	0.40
				T-C-G	85.60%	0.024	0.42
Pore rates	0.028	< 0.1	5.58	SNP11–13			
				T-T-A	7.99%	0.021	9.10
	0.008	< 0.1	10.26	SNP35–37			
				T-T-T	5.60%	0.020	9.00
				G-C-T	74.00%	0.031	10.04
Radial lumen diameter	0.005	< 0.1	12.21	SNP7–9			
				T-T-A	3.97%	0.025	15.10
	0.011	< 0.1	10.08	SNP19–21			
				T-A-T	6.80%	0.014	15.39
	0.006	< 0.1	8.68	SNP23–25			
				C-T-A	42.79%	0.005	16.52
				C-T-C	52.40%	0.031	16.01
	0.012	< 0.1	10.61	SNP25–27			
				A-C-T	42.79%	0.005	16.52
Radial fiber central cavity diameter	0.042	< 0.1	3.40	SNP3–5			
				T-C-T	8.00%	0.016	18.30
	0.046	< 0.1	7.34	SNP8–10			
				T-A-C	3.95%	0.005	17.73
	0.009	< 0.1	10.58	SNP19–21			
				T-A-T	6.80%	0.041	18.41
				C-A-A	79.89%	0.032	19.17
	0.005	< 0.1	9.08	SNP23–25			
				C-T-A	42.79%	0.013	19.33
Chordwise lumen diameter	0.035	< 0.1	7.89	SNP7–9			
				T-T-A	3.97%	0.021	12.71
Chordwise fiber central cavity diameter	0.015	< 0.1	7.24	SNP7–9			
				T-T-A	3.97%	0.002	15.35
	0.003	< 0.1	5.15	SNP14–16			
				T-A-T	5.20%	0.006	15.60
Average fiber central cavity diameter	0.002	< 0.1	11.45	SNP7–9			
				T-T-A	3.97%	0.001	16.54
	0.032	< 0.1	3.21	SNP14–16			
				T-A-T	5.20%	0.011	16.94

*P value* significance of the haplotype-based association (*P* ≤ 0.05), *Q value* correction for multiple testing (false discovery rate, Q ≤ 0.10), *R*^*2*^ percentage of the phenotypic variance explained

## Discussion

### Putative function of *CfSUS*

*SUS* is an important enzyme participated in cellulose synthesis, which is the major component of plant cell walls. Li et al. speculated that *SUS* was associated with juvenile wood density in *Pinus radiata* [25], which is an important trait of timber from many species. Thus, numerous studies have focused on *SUS* genes, including the identification of 15 *SUS* genes in the *Populus trichocarpa* genome [26]. In this study, we cloned the full-length *CfSUS* cDNA from *Catalpa fargesii* and further constructed a phylogenetic tree using 13 deduced amino acid sequences of *SUS* from *Arabidopsis thaliana* and *P. trichocarpa*, and the results indicated that *CfSUS* is an ortholog of *AtSUS*1,4 and *PtrSUS*1,2 (Additional file 8: Figure S4). Down-regulation of *SUS*1 and *SUS*2 in *Populus* negatively influence wood density, cell wall thickness, and other anatomical parameters, ultimately decreasing the wood stiffness and ultimate stress [27]. The phylogenetic analysis indicated that *CfSUS* may have a similar function to *PtrSUS*1 and *PtrSUS*2 and may be associated with important wood mechanical properties in *C. fargesii*; however, this remains to be clarified. In the present study, relatively high expression was observed in xylem compared with phloem and leaf, similar to the expression pattern of *SUS*1 in *Populus tomentosa* [8]. These results indicated that *CfSUS* may be involved in cellulose biosynthesis in secondary xylem and may be associated with wood density and anatomical parameters according to a previous study [27].

### Sequence polymorphisms and LD estimation of *CfSUS*

SNP-based association analyses are important for elucidating the SNP distribution and frequency patterns within a candidate gene [28]. In this study, *CfSUS* was chosen to perform sequencing-based SNP discovery and analysis. The nucleotide polymorphism rates of the total sequence, exon and intron regions of *CfSUS* were 3.85%, 3.13%, and 5.36%, respectively (Table 1). Exons had a lower nucleotide polymorphism rate than introns, which is in accordance to other studies [6, 24], indicating that exon sequences may be more conserved than intron sequences under selective pressure. Compared to our another study on nucleotide polymorphism of a *C3h* gene in *Catalpa fargesii* using nearly same mapping population (in that study, 88 *C. fargesii* were randomly selected from the same 144 individuals as mapping population and most of which were also selected in this study), the nucleotide diversity (*π*_T_ = 0.0034) of *CfSUS* was similar to that of *CfC3h* (*π*_T_ = 0.0031, unpublished data), suggesting that these two genes share a similar pattern of genetic variance in natural *C. fargesii* populations. However, the nucleotide diversity of *CfSUS* was lower than that of *PtSUS*1 (*π*_T_ = 0.0092) and *PtSUS*2 (*π*_T_ = 0.0109), which had been proved as potential homologous gene in present study in *Populus tomentosa* [8]. Our result indicated that the *SUS* gene may be more conserved in *C. fargesii* than *P. tomentosa.*

Understanding LD patterns is indispensable for association mapping. In our study, LD in *CfSUS* was rapidly decayed within 1600 bp (r^2^ < 0.1; Fig. 2), supporting the use of association mapping to identify genes, and even SNPs, responsible for variations in traits [29]. Our results were basically in accordant to those of LD studies on *P. tomentosa* [30], *Eucalyptus nitens* [31], *Pinus sylvestris* [32], loblolly pine [33], and Douglas fir [34], which showed a similar rapid decay in LD. The low LD was generally found in forest trees, both deciduous and coniferous trees, may be due to the large effective population sizes, tendency for outcrossing, and a long history of recombination [35]. Moreover, *CfSUS* was similar to *CfC3h* (unpublished data, with the r^2^ dropping below 0.1 within 1.8 kb), which indicated that low LD may also existed in other genes of *C. fargesii*. However, few studies have assessed the LD in *C. fargesii*, and the degree of LD in other genes or the whole genome of *C. fargesii* is unknown. In future studies, we will estimate LD decay in more genes and longer genomic fragments and even explore the haplotype variability on the whole-genome level.

Five distinct haplotype blocks identified within the *CfSUS* gene (Fig. 3) according to the LD analysis using 47 common SNPs from 125 individuals in the association population. The blocks were small with SNP markers in each distinct haplotype block being close to one another, which was consistent with the rapid decay in LD of *CfSUS* gene. Overall, the low LD observed in the *CfSUS* gene is indicative of a high resolution of marker–trait associations.

### Association mapping of wood properties

Based on association mapping of wood quality traits, we identified 17 significant associations representing 11 SNPs in the association population based on the association mapping of the traits (Table 2). Most of the associations identified in this study explained a small proportion of the tested phenotypic variance and was in accordance to other association analysis on wood quality trait in other forest trees [36, 37], which may because wood property associated traits are usually quantitative and influenced by multi-SNP alleles variances in functional genes, all the small effects were attributed to final individual phenotypic variation [38]. In previous studies, many significant associated SNPs have been proven to be nonsynonymous, possibly due to alterations in amino acids. However, in this study, only four associations involving three SNPs (SNP 9, SNP 30, and SNP 34) were nonsynonymous and the other eight significant SNPs were located in introns (four SNPs) or were synonymous (four SNPs). These results were similar to those of Tian et al. [30], in which only a small percentage of SNPs significantly associated with trait variance in *PtoCesA*7 were nonsynonymous mutations. Although synonymous mutations or mutations in introns and non-coding regions do not cause amino acid changes, they can result in the variances through other ways. For example, mutations in 5’UTR and introns may affect gene expression and efficiency of transcript splicing [6]. Synonymous mutations in exons associated with wood properties have been identified in other studies. For example, a synonymous SNP in an exon of *PtoCesA4* was associated with holocellulose content in *Populus tomentosa* [2]. In addition, nucleotide mutations in the 3′UTR could affect mRNA deadenylation and degradation [39].

Wood density together with other traits, such as cellulose microfibril angle determines the wood stiffness of forest trees [25]. In this study, five SNPs were significantly associated with wood density, and the identified associations explained 5.11–6.39% of the wood density variance. However, they were not supported by haplotype association. If a single SNP marker associated to the same trait with the haplotype surrounding it, which would indicate that this SNP probably located near or exactly be the functional variance [40]. In our study, several haplotype-based and single-SNP-based associations containing a common SNP were significantly associated with pore rate (SNP 37), radial lumen diameter (SNP 23), chordwise lumen diameter (SNP 7, 8, and 16), radial central diameter (SNP 23), and chordwise central diameter (SNP 7, 8, and 16) in the association population. All five SNPs were located in intron regions or synonymous mutations. This was consistent with Porth et al. [41], who found that most SNPs significantly associated with traits were located in noncoding regions. The haplotype from block SNP 7–9 was associated with both chordwise lumen diameter and chordwise central diameter, explaining 7.89% and 7.24% of their phenotypic variation, respectively, slightly higher than that of SNP 7 but lower than SNP 8, which indicated that markers around SNP 7 and 8 may interact with these two loci and contribute to phenotypic effects. In addition, both of the SNPs were located in an intron region and may affect phenotypic variations by influencing RNA splicing [6]; however, the detailed mechanisms require further investigation.

Our study indicated that *CfSUS* was associated to wood properties and could be considered as a candidate gene for future marker-assisted breeding in *C. fargesii*. However, we only made the association study of *CfSUS* and the formation of wood properties is a very complex process that require the coordinate regulation of multiple genes. To better understand the genetic variance of the wood properties associated traits, association studies on the whole genome wide level of *C. fargesii* based on the re-sequencing technology would be considered in our future studies.

## Conclusion

In this study, we cloned a *CfSUS* gene that share a high sequence similarity to other *SUS* genes in *C. fargesii* and identified 135 SNPs through amplifying and sequencing the same locus from 93 individuals a mapping population. Moreover, LD did not extend over the entire gene (r^2^ < 0.1, within 1600 bp), which indicated that *CfSUS* have a potential utility of a gene-based association mapping method in developing SNP markers in *C. fargesii*. Finally, we identified 11 SNPs and 14 haplotypes were significantly associated with wood property by association analysis. These findings imply a functional role of *CfSUS* in mediating wood properties and providing SNP markers associated with wood property, suggesting the potential application in marker-assisted breeding of *C. fargesii* in the future.

## Additional files

Additional file 1 **Table S1.** Phenotype of associated population. (XLSX 19 kb)

Additional file 2 **Figure S1.** The distribution pattern for each trait measured in the entire population (*n* = 125) of *Catalpa fargesii*. (JPG 7743 kb)

Additional file 3 **Table S2.** The mean values, range of variation (RV), standard error (SE) and coefficient of phenotypic variation (CV (%)) for each wood property trait measured in association population. (DOCX 18 kb)

Additional file 4 **Table S3.** Primers used in this paper. (DOCX 15 kb)

Additional file 5 **Table S4.** Genotypic data of *CfSUS* identified in the associated population. (XLSX 30 kb)

Additional file 6 **Figure S2.** Genomic organization of *CfSUS*. Positions of 47 common SNP markers are shown as vertical lines. (JPG 1318 kb)

Additional file 7 **Figure S3.** Phenotypes of different genotypes of significant SNPs associated with wood property traits. (JPG 6446 kb)

Additional file 8 **Figure S4.** An unrooted phylogenetic tree of *SUS* members from *Arabidopsis thaliana*, *Populus trichocarpa* and *Catalpa fargesii*. *Arabidopsis thaliana*: *ATSUS*1(AT5G20830.1), *ATSUS*2(AT5G49190), *ATSUS*3(AT4G02280), *ATSUS*4(AT3G43190), *ATSUS*5(AT5G37180) and *ATSU*S6(AT1G73370); *Populus trichocarpa*: *PtrSUS*1(Potri.018G063500.1), *PtrSUS*2(Potri.006G136700.1), *PtrSUS*3(Potri.002G202300.1), *PtrSUS*4(Potri.015G029100.1), *PtrSUS*5(Potri.012G037200.1), *PtrSUS*6(Potri.004G081300.1), *PtrSUS*7(Potri.017G139100.1); *Catalpa fargesii* Bur.:*CfSUS*. (JPG 1588 kb)

## Abbreviations

CDS: Complete coding sequence
LD: Linkage disequilibrium
MLM: Mixed linear model
ORF: Open reading frame
SNP: Single-nucleotide polymorphism
*SUS*: Sucrose synthase
WBD: Wood basic density
