# Supplementary figures and images for: Single-nucleotide polymorphisms(SNPs) in a sucrose synthase gene are associated with wood properties in Catalpa fargesii bur
Source: BMC Genet. 2018 Nov 1;19:99. doi: 10.1186/s12863-018-0686-8 (PMC6211571; doi:10.1186/s12863-018-0686-8)

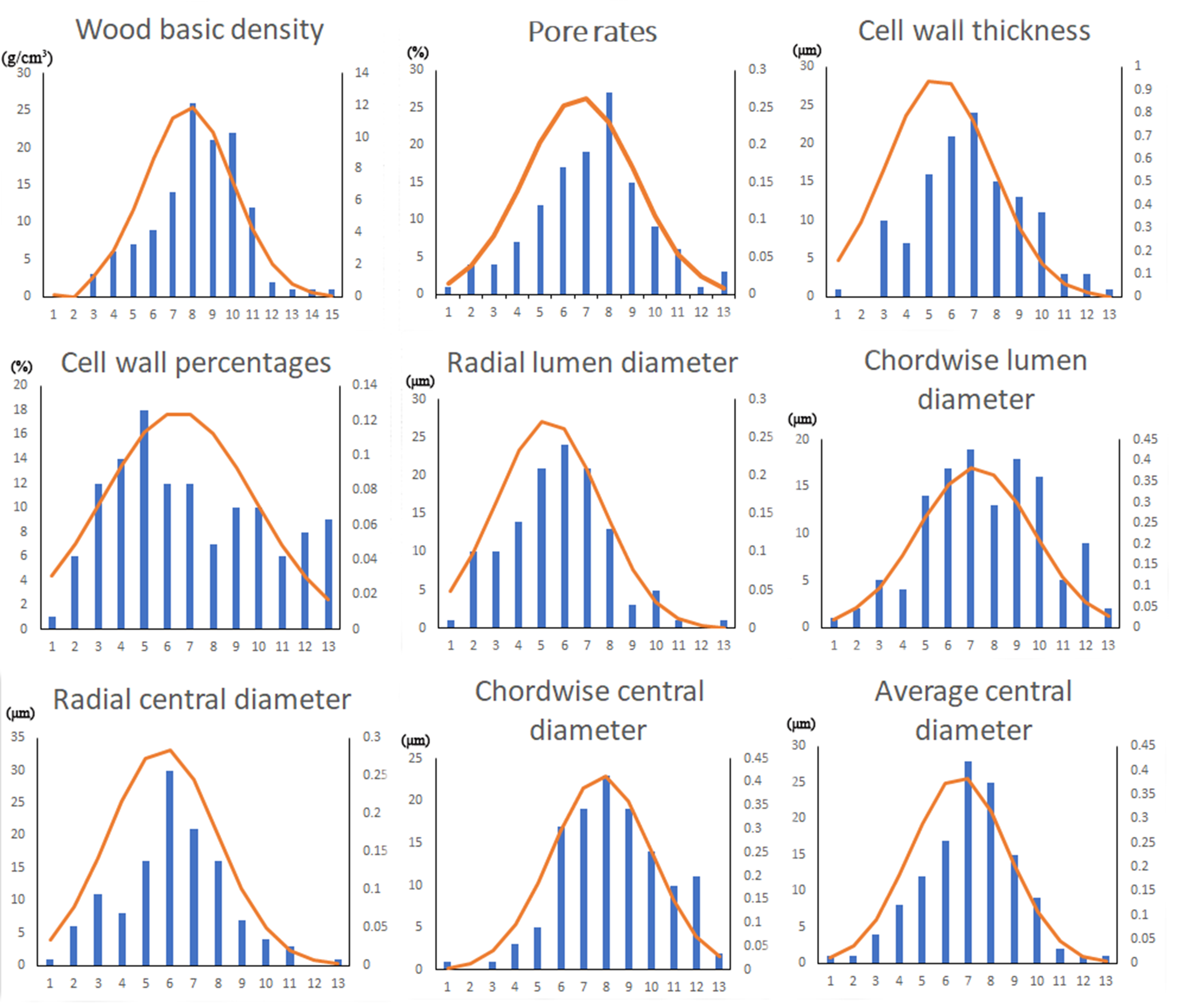

Supplement: Supplementary file 2 — Figure S1. The distribution pattern for each trait measured in the entire population (n = 125) of Catalpa fargesii. (JPG 7743 kb) [file 12863_2018_686_MOESM2_ESM.jpg]

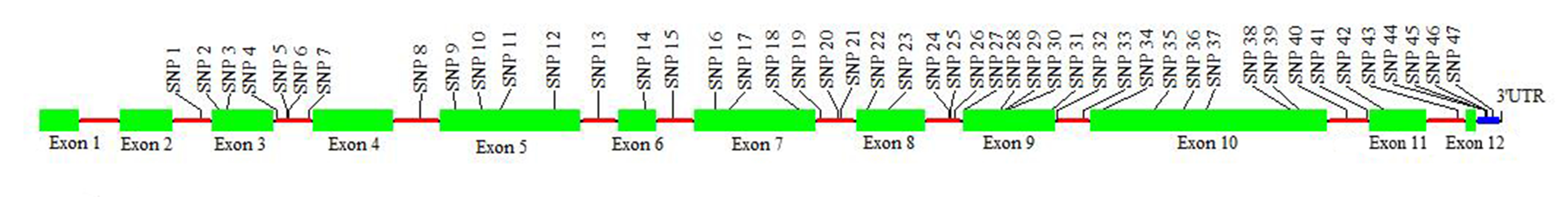

Supplement: Supplementary file 6 — Figure S2. Genomic organization of CfSUS. Positions of 47 common SNP markers are shown as vertical lines. (JPG 1318 kb) [file 12863_2018_686_MOESM6_ESM.jpg]

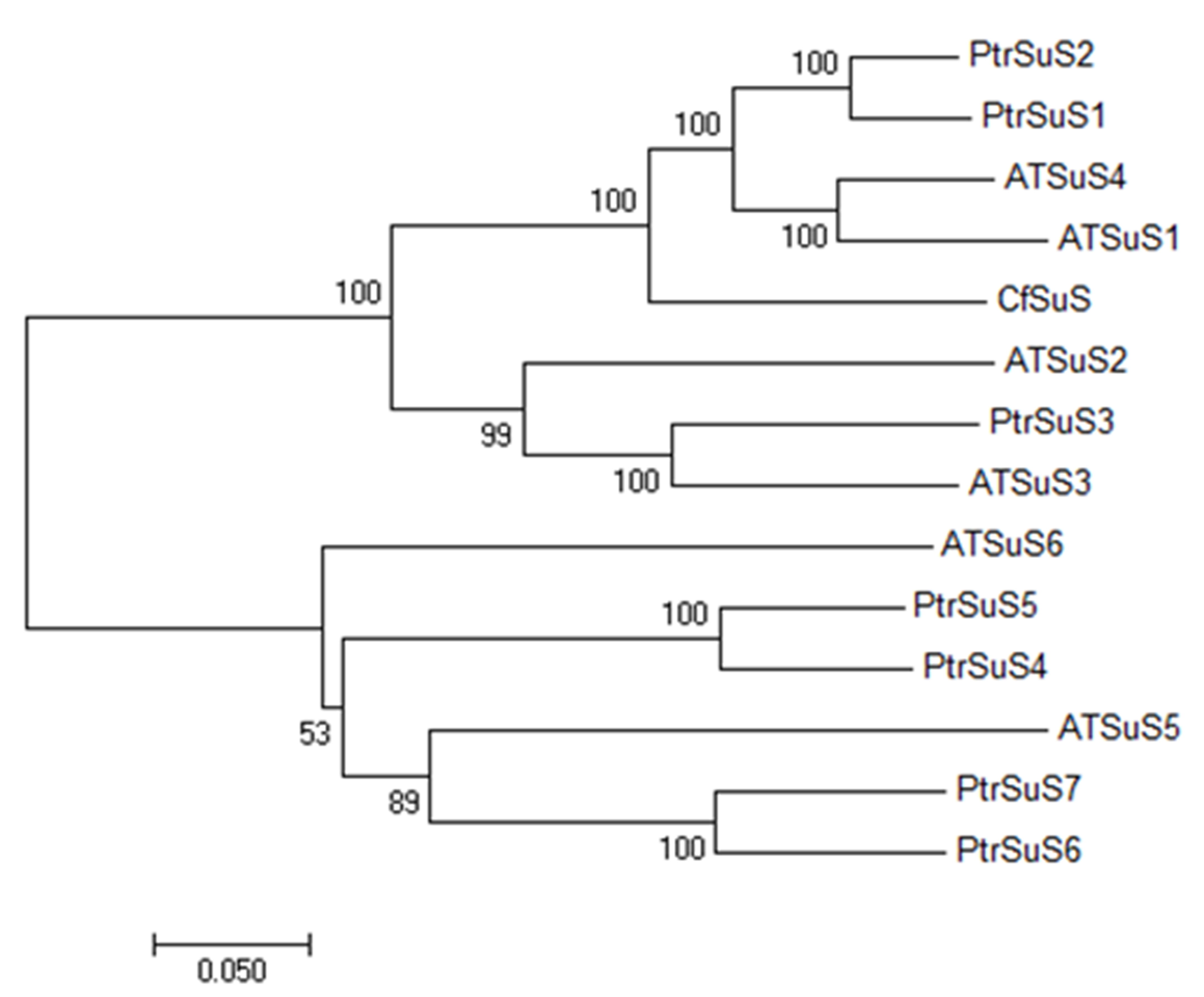

Supplement: Supplementary file 8 — Figure S4. An unrooted phylogenetic tree of SUS members from Arabidopsis thaliana, Populus trichocarpa and Catalpa fargesii. Arabidopsis thaliana: ATSUS1(AT5G20830.1), ATSUS2(AT5G49190), ATSUS3(AT4G02280), ATSUS4(AT3G43190), ATSUS5(AT5G37180) and ATSUS6(AT1G73370); Populus trichocarpa: PtrSUS1(Potri.018G063500.1), PtrSUS2(Potri.006G136700.1), PtrSUS3(Potri.002G202300.1), PtrSUS4(Potri.015G029100.1), PtrSUS5(Potri.012G037200.1), PtrSUS6(Potri.004G081300.1), PtrSUS7(Potri.017G139100.1); Catalpa fargesii Bur.:CfSUS. (JPG 1588 kb) [file 12863_2018_686_MOESM8_ESM.jpg]
