# Supplementary material for: Single-nucleotide polymorphisms(SNPs) in a sucrose synthase gene are associated with wood properties in Catalpa fargesii bur
Source: BMC Genet. 2018 Nov 1;19:99. doi: 10.1186/s12863-018-0686-8 (PMC6211571; doi:10.1186/s12863-018-0686-8)
Supplement: Supplementary file 3 — Table S2. The mean values, range of variation (RV), standard error (SE) and coefficient of phenotypic variation (CV (%)) for each wood property trait measured in association population. (DOCX 18 kb) [file 12863_2018_686_MOESM3_ESM.docx]

Table S2 The mean values, range of variation (RV), standard error (SE) and coefficient of phenotypic variation (CV (%)) for each wood property trait measured in association population

| Traits | Mean values | RV | SE | CV (%) |
| --- | --- | --- | --- | --- |
| Wood basic density（g/cm^3^） | 0.42 | 0.33~0.49 | 0.03 | 7.30 |
| Pore rates（%） | 9.93 | 7~14 | 1.55 | 15.57 |
| Cell wall percentages（%） | 35.22 | 22.05~41.62 | 3.57 | 10.12 |
| Cell wall thickness（μm） | 2.85 | 1.52~3.85 | 0.46 | 16.01 |
| Radial lumen diameter（μm） | 16.25 | 13.45~20.45 | 1.40 | 8.61 |
| Radial fiber central cavity diameter（μm） | 13.42 | 11.15~15.88 | 0.98 | 7.28 |
| Chordwise lumen diamete  （μm） | 19.09 | 15.43~23.03 | 1.34 | 7.04 |
| Chordwise fiber central cavity diameter  （μm） | 16.26 | 12.68~18.5 | 0.95 | 5.82 |
| Average fiber central cavity diameter  （μm） | 17.66 | 14.05~20.38 | 1.02 | 5.80 |

Table S2 Part of primers used in this paper

| Name | Function | Sequence | Tm | Length |
| --- | --- | --- | --- | --- |
| SUS-CDS | CDS amplification | 5'-ATCCCTCTGTTCGCCATTTC-3' | 60℃ | 2418 bp |
|  |  | 5'-TCACTCAGCAGCCAGTGGAACA -3 |  |  |
| SUS-a | Intron amplification | 5' ATCCCTCTGTTCGCCATTT 3'  5' GCGTCTCAGGAGGAAGTGT 3' | 53℃ | 2190 bp |
| SUS-b | Intron amplification | 5' TGGGTTGCGCTTGCTATTC 3'  5' CTTCGATTTCAGGGTGGAG 3' | 53℃ | 1949 bp |
| SUS-c | Intron amplification | 5' CCCCAAATTCAACATCGT 3'  5' AGTTCACCGTTCCTCACA 3' | 48℃ | 417 bp |
| SUS-d | Intron amplification | 5' TGGATTATACCGGGTTGTC 3'  5' AGGTAGCATTACATGGGTC 3' | 51℃ | 1260 bp |
| SUS-e | Integrality verification | 5' ATCCCTCTGTTCGCCATTTC 3'  5' AGCCTGTTCTTCCCGCAATA 3' | 53℃ | 4787bp |
| SUS-1 | Gene amplification | 5'- CCTTAAAAGTCATCTTCA -3'  5'-AGCATAAAGTCCTTGTCGC-3' | 44℃ | 998 bp |
| SUS-2 | Gene amplification | 5'-TCCACAGACAATGATGCTGAATG-3'  5'-CCCTCGCTATAGTTGCCAATAAT-3' | 51.6℃ | 853 bp |
| SUS-3 | Gene amplification | 5'-GAACCGAGAAGGGCATTG-3'  5'-GGGACCACCGTAAAGAGT-3' | 51℃ | 1247 bp |
| SUS-4 | Gene amplification | 5'-CCTTGTCGTAGTTGGTGG-3'  5'-TGTTCTTCCCGCAATATT-3' | 48℃ | 849 bp |
| SUS-q | RT-qPCR | 5’-ATCTTGCGGCGTCGTTGCTT-3’ | 60℃ | - |
|  |  | 5’-TCCATATCATCCCAGTTGCT-3’ |  |  |
| Actin | Internal control | 5’-GATGATGCTCCAAGAGCTGT-3’ | 55℃ | - |
|  |  | 5’-TCCATATCATCCCAGTTGCT-3’ |  |  |
