# Supplementary material for: Single-nucleotide polymorphisms(SNPs) in a sucrose synthase gene are associated with wood properties in Catalpa fargesii bur
Source: BMC Genet. 2018 Nov 1;19:99. doi: 10.1186/s12863-018-0686-8 (PMC6211571; doi:10.1186/s12863-018-0686-8)
Supplement: Supplementary file 4 — Table S3. Primers used in this paper. (DOCX 15 kb) [file 12863_2018_686_MOESM4_ESM.docx]

Table S3 Primers used in this paper

| Name | Function | Sequence | Tm | Length |
| --- | --- | --- | --- | --- |
| SUS-CDS | CDS amplification | 5'-ATCCCTCTGTTCGCCATTTC-3' | 60℃ | 2418 bp |
|  |  | 5'-TCACTCAGCAGCCAGTGGAACA -3 |  |  |
| SUS-a | Intron amplification | 5' ATCCCTCTGTTCGCCATTT 3'  5' GCGTCTCAGGAGGAAGTGT 3' | 53℃ | 2190 bp |
| SUS-b | Intron amplification | 5' TGGGTTGCGCTTGCTATTC 3'  5' CTTCGATTTCAGGGTGGAG 3' | 53℃ | 1949 bp |
| SUS-c | Intron amplification | 5' CCCCAAATTCAACATCGT 3'  5' AGTTCACCGTTCCTCACA 3' | 48℃ | 417 bp |
| SUS-d | Intron amplification | 5' TGGATTATACCGGGTTGTC 3'  5' AGGTAGCATTACATGGGTC 3' | 51℃ | 1260 bp |
| SUS-e | Integrality verification | 5' ATCCCTCTGTTCGCCATTTC 3'  5' AGCCTGTTCTTCCCGCAATA 3' | 53℃ | 4787bp |
| SUS-1 | Gene amplification | 5'- CCTTAAAAGTCATCTTCA -3'  5'-AGCATAAAGTCCTTGTCGC-3' | 44℃ | 998 bp |
| SUS-2 | Gene amplification | 5'-TCCACAGACAATGATGCTGAATG-3'  5'-CCCTCGCTATAGTTGCCAATAAT-3' | 51.6℃ | 853 bp |
| SUS-3 | Gene amplification | 5'-GAACCGAGAAGGGCATTG-3'  5'-GGGACCACCGTAAAGAGT-3' | 51℃ | 1247 bp |
| SUS-4 | Gene amplification | 5'-CCTTGTCGTAGTTGGTGG-3'  5'-TGTTCTTCCCGCAATATT-3' | 48℃ | 849 bp |
| SUS-q | RT-qPCR | 5’-ATCTTGCGGCGTCGTTGCTT-3’ | 60℃ | - |
|  |  | 5’-TCCATATCATCCCAGTTGCT-3’ |  |  |
| Actin | Internal control | 5’-GATGATGCTCCAAGAGCTGT-3’ | 55℃ | - |
|  |  | 5’-TCCATATCATCCCAGTTGCT-3’ |  |  |
